# Diversity of the Monstrilloida (Crustacea: Copepoda)

**DOI:** 10.1371/journal.pone.0022915

**Published:** 2011-08-10

**Authors:** Eduardo Suárez-Morales

**Affiliations:** El Colegio de la Frontera Sur Unidad Chetumal, Chetumal, Quintana Roo, Mexico; Institute of Marine Research, Norway

## Abstract

Monstrilloid copepods are protelean parasites of different groups of marine benthic invertebrates. Only their first naupliar, preadult, and adult phases are planktonic. Monstrilloids are currently represented by more than 115 nominal species contained in four genera. Its taxonomic knowledge has been hampered by nomenclatural and descriptive problems derived from their peculiar ontogeny and poor definitions of taxa. One of the most important difficulties is that of matching males to females. The only reliable methods to link the sexes of a species are the confirmation of particular apomorphies shared by both sexes, finding both sexes in the same host or as a pre-copulatory male-female pair in the plankton, or by the use of molecular markers. A general overview of the morphology of the group and its life cycle is provided herein. Recently, upgraded descriptive standards have been established and the relevance of redescribing taxa based on type and museum specimens has been demonstrated. The rate of species description per decade has had several peaks between 1840 and 2010: (1971–1980, 1991–2000, 2001–2010), each related to the activity of a few researchers. An analysis of the world distribution of published records of the Monstrilloida revealed that the Northeast Atlantic is the best studied region (45% of all records), followed by the Northwestern Atlantic (17%); the least surveyed areas include regions of the southern hemisphere (less than 3%). The Northeast Atlantic region harbors the highest number of known species (32 nominal species), followed by the Caribbean Sea/Gulf of Mexico (24), the Mediterranean/Black Sea (19), Indonesia-Malaysia-Philippines region (17), Japanese waters (17), and the Brazil-Argentina area (16). Other than these generalized patterns, little can be concluded concerning the biogeography of the group. Many species records are doubtful or improbable, and purportedly cosmopolitan nominal species are being revealed as species complexes yet to be studied.

## Introduction

The order Monstrilloida Sars, 1901 represents one of the most intriguing taxa among the Copepoda. They are endoparasites of marine invertebrates during their postnaupliar and juvenile stages but also have three free-living phases, an infective naupliar stage, a final copepodite stage that leaves the host but soon moults, and non-feeding adults lacking mouthparts [1], [2]. In general, they are rare; a reduced number of specimens can be obtained occasionally during plankton samplings from shallow coastal environments, particularly at night [3]. There are, however, reports of relatively high local concentrations of monstrilloids in reef-related areas [4], [5], where they can be highly diverse [6].

**Figure 1:**
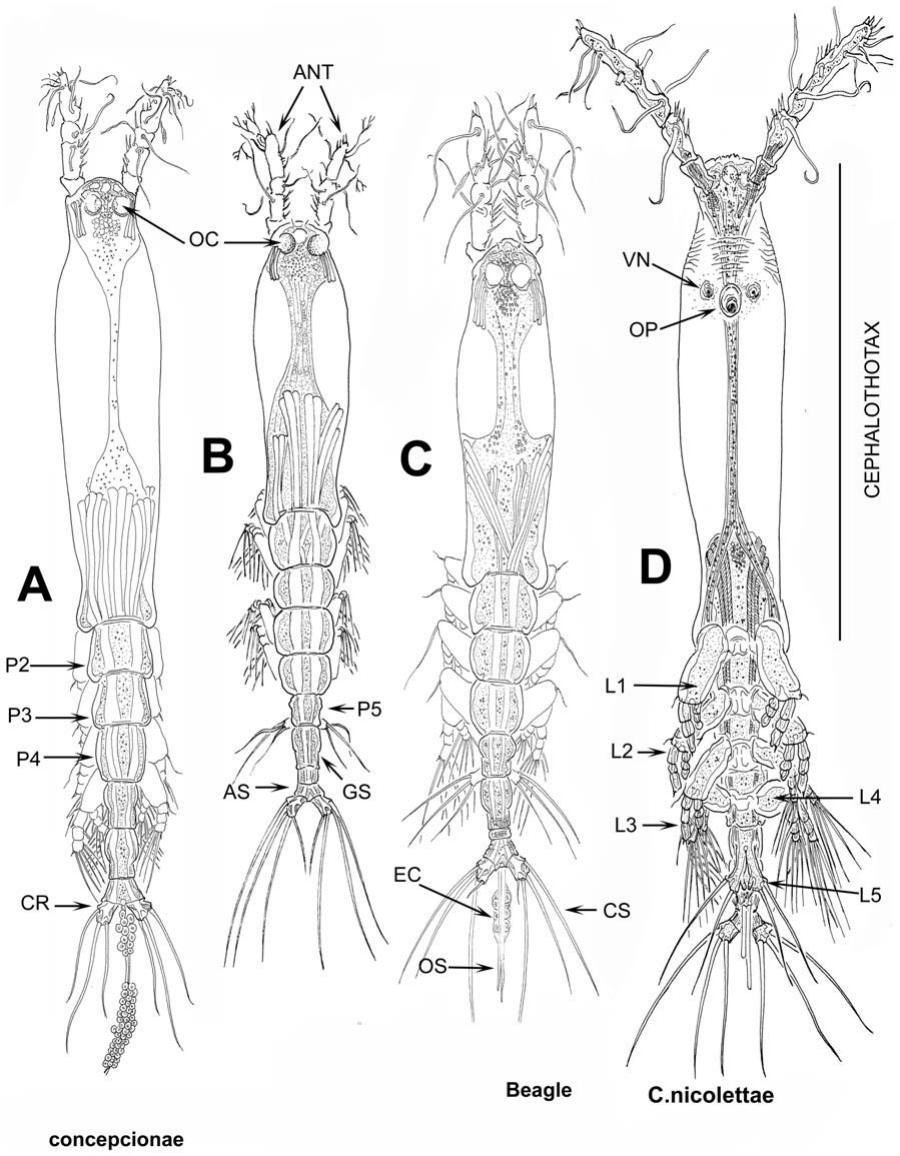
Generalized body plan and different body shapes of female Monstrilloida. A. *Cymbasoma cocoense* Suárez-Morales & Morales-Ramírez, 2009, habitus, dorsal view; B. *Monstrillopsis chilensis* Suárez-Morales et al. 2008, dorsal view; C. *Monstrillopsis igniterra* Suárez-Morales et al. 2008, dorsal view; D. *Cymbasoma nicolettae* Suárez-Morales, 2002, ventral view. ANT = antennules; AS = anal somite; CR =  caudal ramus; CS = caudal seta; EC = egg cluster; GS = genital somite; L1–L5 = legs 1–5; OC = ocellus; OP = oral papilla; OS = ovigerous spine; P2–P5 = pedigerous somites 2–5; VN = ventral nipple-like process.

**Figure 2:**
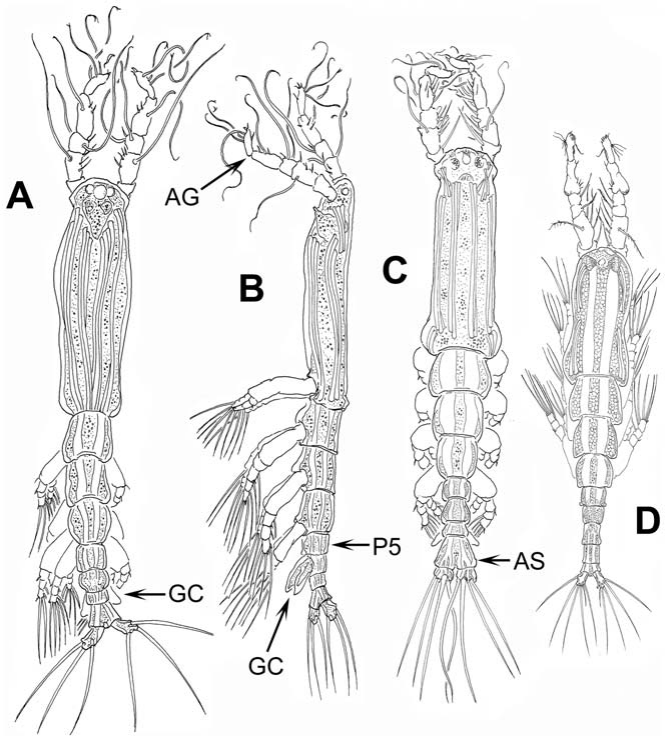
Generalized body plan and different body shapes of male Monstrilloida. A. *Cymbasoma quadridens* Davis, 1947, dorsal view; B. same, lateral view, C. *Cymbasoma bullatum* (A. Scott, 1909) [30], dorsal view; D. *Monstrilla patagonica* Suárez-Morales et al., 2008, ventral view. AG = antennule geniculation, GC = genital complex.

As a group, monstrilloids have been observed mainly from the earliest marine planktological surveys carried out during the 19^th^ century. Most of the earliest descriptions were from the Northeast Atlantic and the Mediterranean. The first record of a monstrilloid copepod was published by Krøyer in 1842 [7], who illustrated a single specimen from a Norwegian fjord. He named it *Thaumatoessa typica* Krøyer, but with no descriptive text accompanying the figure. A description was provided by him later but with a change of name to *Thaumaleus typicus* Krøyer, 1849 and a diagnosis of this new nominal genus [8]. The original specimen was re-examined by Grygier in 1994 [9], who determined that this first described monstrilloid is in fact a preadult (i.e. last stage copepodite larva) specimen that probably belongs to a species of the genus *Monstrilla*. *Thaumaleus* is thus now a junior subjective synonym of *Monstrilla*, but *Monstrilla* in turn became a subjective junior synonym of *Thaumatoessa* [6], [9]. To conserve *Monstrilla*, *Thaumatoessa* was suppressed by the International Commission on Zoological Nomenclature. Both generic names proposed by Krøyer include the Greek root *thaumato*, meaning wondrous thing, miracle. Monstrilloids no doubt attracted the attention of anatomists and zoologists because of their striking and unusual features: adults have no antennae or mouthparts and their antennules are distinctly straight (except for the distal segment of males) and anteriorly directed (Figures 1, 2). The lack of mouthparts was also explicitly noted by Dana in 1849 [10], who referred to this character as “…*maxillis pedibusve non munitus…*” and this oddity probably inspired his proposal of *Monstrilla* (i.e. little monster) as a generic name for his find from the Sulu Sea, the first Pacific record of this group. Other old, now invalid or uncertain generic names for monstrilloids include also *Haemocera* Malaquin, 1896 and *Thaumatohessia* Giard, 1900 [6]. *Haemocera filogranarum* Malaquin 1901, one of the four species described in this genus (the others being *H. danae*, *H. roscovita*, and *H. ostroumowi*), is probably a species of *Monstrillopsis* [11].With the recent exclusion of *Strilloma* Isaac, 1974 as a valid genus [12], the order Monstrilloida currently includes four valid genera: *Monstrilla* Dana, 1849, *Cymbasoma* Thompson, 1888, *Monstrillopsis* Sars, 1921, and the newest genus *Maemonstrilla* Grygier & Ohtsuka, 2008.

Early studies of the biology of these copepods by French and Dutch researchers [13]–[17] resulted in the realisation that these microcrustaceans lived a double life, endoparasitic and planktonic. Some species were observed as nodules on the mantle of molluscs or swellings of the body surface of polychaetes. From these authors we have descriptions of the endoparasitic phase, the physical placement in the host, and the manner of exiting the host. Adults of *M. helgolandica* Claus were obtained by rearing larval specimens obtained from the gastropod mollusc *Odostomia scalaris* (McGillivray, 1843) [17]. An overview of the current knowledge of the group, with an emphasis on its diversity and taxonomical problems, but also including aspects of its morphology and biogeography, is presented here.

## Analysis

Based on the available literature and worldwide records of monstrilloids obtained from different sources, various aspects of the diversity and distribution of the group were analysed. From this process, it was possible to obtain a closer overview about the current knowledge of this taxon, including some interesting points of its historical development, i.e. rate of species description since the first species was discovered, its biogeography, the estimated species diversity, and the proportion of described males and females in each of the known genera.

## Results and Discussion

### Current knowledge

#### Rate of species description

**Figure 3:**
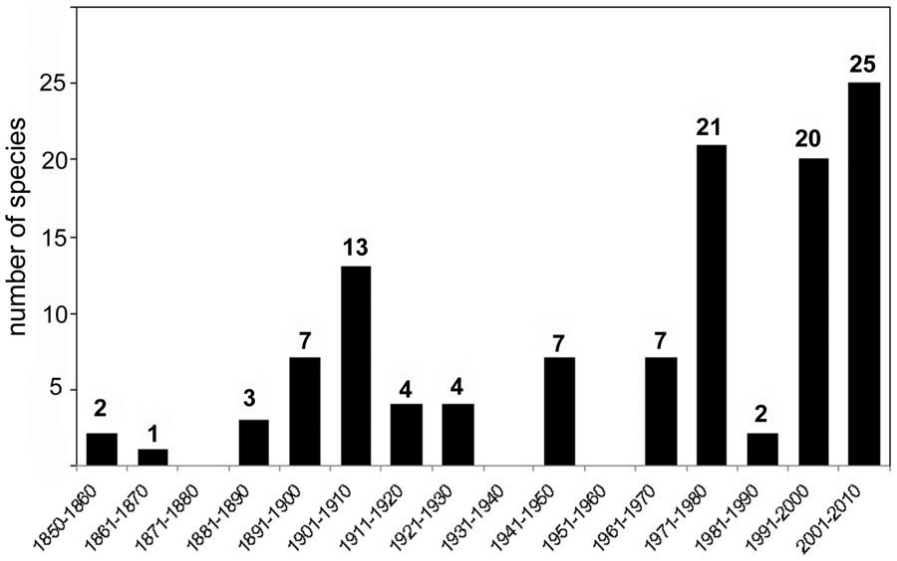
Decadal rate of species descriptions among the Monstrilloida.

The rate of species descriptions per decade (Figure 3) has been highly variable since the discovery of the group in 1841. There are clear peaks in this historical sequence, the earliest one lasting two decades (1891–1910), a second one occurring in 1970–1980, and a third one comprising the past 20 years. Each of these pulses appears to be related to the activity and interest of a few specialists: T. Scott, G.O. Sars, W. Giesbrecht, and A. Scott during the first peak, W.J. Isaac in the second, and Suárez-Morales and colleagues in the most recent period [2], [6], [18]. According to the new rules of the Fourth Edition of the International Code of Zoological Nomenclature, the names of several species described by Isaac and first proposed in a key [18] is their status as “unpublished” until they are made available by later workers, thus affecting the number of species actually published by W.J. Isaac. The literature related to this group was revised in a benchmark document [8] that includes a chronological account and valuable comments on the publications concerning the Monstrilloida between 1840 and 1995. After 1995 the number of works about the Monstrilloida is around 60; marginal mentions in zooplankton surveys or group listings were not accounted in this figure. The geographic coverage of these publications is very wide; it includes the Northwestern Atlantic, Arctic and Antarctic waters, European waters, the Southwestern Atlantic, and the Eastern Pacific. Useful online sources of information are available [18], [19], which include data on most of the nominal species described to date.

Because of the absence of mouthparts and antennae in the adults and the cryptic larval development, diagnoses of monstrilloid genera were based on a limited number of characters, including the number of urosomites in the female, the position of the oral papilla on the cephalothorax, the number of caudal setae, and the development of the eyes [2], [11], [12], [20]. An anteriorly pointing set of ovigerous spines is distinctive of the genus *Maemonstrilla* [6]. It has long been recognized that the group is in need of revision [21], [22]. Two of the known genera have recently been either revised or newly established partly based on revisions, viz., *Monstrillopsis* [11], [23], and *Maemonstrilla* [6]. Revision of the largest genera, *Monstrilla* and *Cymbasoma*, which together account for about 85% of the known species, remains as a pending task. Overall, the systematic study of monstrilloids has involved classificatory conflicts that became even more complex over the decades with shallow descriptions of new species and dubious records of others based on comparisons with poorly defined taxa.

### Species Diversity

There are about 120 nominal species comprised in the family Monstrillidae [6], but some have been synonymized or are deemed as invalid so the number of valid species is somewhat smaller [18] [Suárez-Morales, unpubl. data]. Based on a revision of the available lists and data [8], [18], [24], [Suárez-Morales, unpubl. data], 116 species are recognized here, of which 56 belong to the genus *Monstrilla*, 41 to *Cymbasoma*, 12 to *Monstrillopsis*, and 7 to the recently erected genus *Maemonstrilla*. The generic assignment or validity of about 10 other nominal species, not included in these figures, is uncertain.

The knowledge of the true species diversity of this peculiar group has advanced slowly because of different factors, their specialized biology and ontology, and because of incomplete descriptions.

1) *Biology and Ontogeny*. Monstrilloids have a protelean life cycle, with most of their larval and all but the last juvenile stages being endoparasitic, usable taxonomic characters are not well differentiated at these stages. Hence, the taxonomy of the group historically has been anchored on the morphology of the free-living adult. This in turn, owing to the lack of antennae and mouthparts, offers also a reduced set of characters compared to most other groups among the Copepoda.

One of the main problems in determining the true diversity of the group has been, and still is, the difficulty of linking males and females of a species as they are mixed with those of other species in the water column and consequently in the plankton samples. This circumstance has given rise to distinct, parallel taxonomies for males and females [25]. In most of the early works in which both genders of a species are described, there is little or no indication of how the two sexes were linked; there is thus no certainty that they really belong to the same species. The unreliable criterion of co-occurrence, i.e., finding them in the same plankton sample, has been the most frequently used in the literature to match males to females of a species, even in areas where more than one species are commonly caught in a single tow [6]. The possibility of linking both genders from co-occurrence increases when a species is known exclusively at one site or inhabits a zone with a reduced number of species; however, in most environments more than one species are present.

Unique, key morphological characters, shared between the sexes, have been used to link them in a few species [26], but in general this is not possible. Copulatory or pre-copulatory pairing behavior could provide an unambiguous clue, but as yet no species description has been based on male/female pairs found “*in copulo*” or in “*amplexus*”. Another reliable way to match the male to the female of a species may be to find them emerging from the same host, but only if the latter is assumed to be infected by just a single species of monstrilloid.

A new approach, recently available, is to use molecular and genetic markers to match males to females; these techniques have proved to be useful in distinguishing species of copepods, a genetic profile must be obtained from relatively fresh specimens fixed in ethanol. It is thus useful mainly for recently described species, but the re-examination and redescription of old type and museum specimens, fixed and preserved in formalin, and unusable for genetic analysis, must rely on morphological characters only. Careful morphological examination has indeed provided interesting results and helped to disentangle some of the taxonomic problems at the genus and species levels [9], [12], [27]–[30].

**Figure 4:**
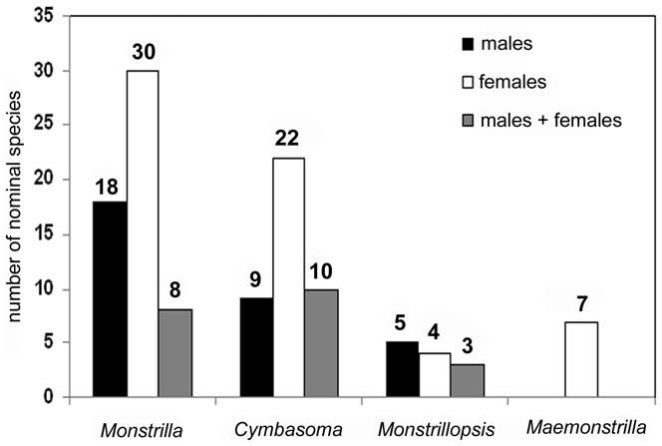
Proportion of described males: females of nominal species in each of the four known genera of the Monstrilloida.

A final option is to try to make new collections for molecular analysis at or near type localities, emphasizing supposedly cosmopolitan species or acknowledged species-groups such as *Cymbasoma rigidum*, *C. longispinosum*, or *Monstrilla helgolandica*, among others. In the ideal situation, we could thus obtain a more accurate vision of the genetic diversity of the group, and of course males and females of species could be spotted and re-grouped. Overall, there are currently 63 nominal species known from females only, 32 from males, and both genders are described for 21 species only, with the caveats mentioned above. Figure 4 shows the known gender distribution among the four valid genera; only *Maemonstrilla* is known exclusively from females, owing to the mostly brooding-related characters.

2) *Incomplete early descriptions*. This is clearly a problem that affects many groups of marine invertebrates, including the Copepoda. Among the Monstrilloida, most of the first descriptions were shallow and illustrations were, in general, poor. There were, however, fine examples of the contrary, as in the works by W. Giesbrecht and G.O. Sars. The problems derived from incomplete descriptive works became more serious over the decades, when species were recorded in different geographic regions based on a general resemblance to these uninformative drawings or shallow descriptions. One such case is *Cymbasoma rigidum* Thompson, 1888, recorded over the decades from many different coastal areas throughout Europe and the Americas; this species is known to contain at least four distinct taxa and most records should be revised [31]. Another example in the same genus is the *C. longispinosum* (Bourne, 1890) species group [32]; this nominal species was presumed to have a cosmopolitan distribution [20]. It is currently known to contain five species that have subtle but consistent differences and are distributed in distinct geographical areas, including Europe (*C. longispinosum*), the Gulf of Mexico (*C. chelemense* Suárez-Morales & Escamilla, 1997), the Gulf of California (*C. californiense* Suárez-Morales & Palomares-García, 1999), Japan, Vietnam, and India (*C. morii* Sekiguchi, 1982 [27]), and the Red Sea, Egypt (*C. janetae* Mageed, 2010). A recent record of *C. longispinosum* from Brazil [33] could also refer to an undescribed taxon of this species-group. In the genus *Monstrillopsis*, the once purportedly cosmopolitan *M. dubia* (T. Scott, 1904) is now known to contain at least three different species [23]. Within the genus *Monstrilla*, *M. helgolandica* is also deemed as a cosmopolitan species, but some records are morphologically distinct [34]. To solve such problems it is urgent to increase efforts to revise and standardize the morphological knowledge of the nominal species by recovering type specimens and re-examining them, or by redescribing species based on neotypes if required, all according to recent, upgraded standards [6], [34]. Out of the 116 nominal species recognized as valid, only 24 of them (21%) have been redescribed or revised in the past 20 years based on type or museum specimens.

### Classification, phylogeny

Monstrilloids were at first included as part of the Order Entomostraca and within the Suborder Cormostomata, which contained four tribes (equivalent to the family level), one of which was named Monstrillacea (now Monstrillidae) and contained a single genus and species, *Monstrilla viridis* [10]. After this first classification, monstrillids were come to be regarded as a sub-order of the Copepoda, occasionally being classified together with the Thespesiopsyllidae. This latter group contains a few species that share with monstrillids the absence of mouthparts and a protelean life cycle. The suborder Monstrilloida was accordingly split into two sections, the Monstrilloida Cyclopimorpha containing the family Thespesiopsyllidae, and the Monstrilloida Genuina, comprising the family Monstrillidae [35]. Thespesiopsyllids were later removed from the Monstrilloida based on their clear affinities with the Cyclopoida [22], [36] as is shown by their possession of typical cyclopoid characters such as the presence of egg sacs (absent in the Monstrilloida) and a median copulatory pore with lateral oviducal openings (vs. fused with a median aperture in Monstrilloida). Much later, this reduced group was then recognized as representative of a new order of the Copepoda, the Thaumatopsylloida, with one genus and five species [37]. The phylogenetic position of the Monstrilloida among the Copepoda was also analysed and discussed by Huys and Boxshall [22]; they stated that, despite the major obstacle posed by lack of mouthparts, the Monstrilloida were tentatively positioned close to the Siphonostomatoida, as a sister taxon. Both groups share a cephalothorax fully incorporating the first pedigerous somite, and a similar structure of the fifth leg. However, these characters have also been dismissed as evolutionary convergences that are known to occur in other copepod orders as well [38], thus providing no reliable base upon which to postulate a common ancestry. The problem of the lack of comparative information related to the mouthparts is deeper because the monophyly of the Siphonostomatoida has been defined by characters of such appendages. It has been considered that, otherwise, the monstrilloid body plan offers few morphological clues of phylogenetic significance [38].

Consequently, Huys et al [38] have been forced to restrict their evaluation of morphological characters to the armature of the antennules and the setation of the caudal rami to supplement the characters of the missing cephalic appendages. They used also molecular data from rDNA genes and the validity of the Monstrilloida as an order has been questioned as a result. This group appears to represent a branch of the siphonostomatoids that colonized a different group of hosts but shares a common ancestor with the caligiform families. Overall, there are still relevant characters to revisit and compare, such as the morphology of the appendages of the naupliar stages. Here, unique characters have been observed [34], compared to nauplii of other orders of the Copepoda. Also, the patterns of body and leg segmentation during development should be known better before Monstrilloida is abandoned as an independent order. In any case, monstrilloids are a compact, well defined but intriguing group still posing many questions.

### Morphology

The morphology of monstrilloid copepods has been studied in detail by several authors over the decades, and a first overview of the group following a detailed model description has been provided together with a comparative analysis of the ancestral stages of the appendages [22]. A closer view of the morphology of the group and a proposal of successive upgraded descriptive models was provided by M. J. Grygier and S. Ohtsuka [6], [34]. The regular size of the female monstrilloids (excluding the antennules and caudal setae) ranges between 2 and 3 mm and males are smaller (1.3–1.7 mm) [18], but some species are outside these general ranges. The smallest monstrilloids known are the males of *Monstrilla pygmaea* Suárez-Morales, 2000 (0.43 mm) and *M. minuta* Isaac, 1975 (0.49 mm) [39]. The largest species is *Cymbasoma gigas* (A. Scott, 1909) (8.2 mm) [29].

Monstrilloids indeed have striking characters, most of which are linked to their biology. As endoparasitic nauplii and copepodites, they have paired tube-like nourishing processes that allow them to absorb nutrients from their host; once they are discarded, scars of these processes remain in the adults as nipple-like cuticular processes. Last copepodite and adult members of the group can be recognized by the lack of cephalic appendages other than the antennules in both sexes. The antennules show a characteristic orientation, anteriorly directed in all cases. They also have a distinctive pattern of segmentation and a reduced but generally conservative setation pattern [22], [34]. Monstrilloids lack egg sacks and instead they bear trailing ventral ovigerous spines. These are formed by two slender, spiniform structures whose length is variable in different species; both spines are usually equal or subequal in longitude and have a pointed terminal tip sometimes preceded by a subdistal protuberance, as in *M. hamatapex* [27]. Clusters of eggs are attached to these spines by means of a mucous substance secreted by the terminal part of the oviduct. A variation of this pattern is the recent finding of anteriorly pointing ovigerous spines. This is an adaptation for subthoracic brooding, which among planktonic copepods, occurs uniquely in monstrilloids of the genus *Maemonstrilla* [6]. The ovigerous spines are not fully developed in the preadult stage, they appear as short, corrugated structures as in *M. capitellicola* [40] and also in *M. mariaeugeniae* Suárez-Morales & Islas-Landeros, 1993 [41]. At this stage, the fifth leg is weakly developed, with only two or three outer setae and an inner naked process, probably a bud of a lobe to appear at the final moult [22], [40], [41].

Among the general characters of monstrilloids, the body segmentation is similar to most copepods, with a cephalothorax incorporating the first pedigerous somite. The urosome, including the fifth pediger, is composed of 3 (*Cymbasoma*) or 4 (*Monstrilla*, *Monstrillopsis*, *Maemonstrilla*) somites in the females and 4 in males of *Cymbasoma* or 5 in those of *Monstrilla* and *Monstrillopsis*. The urosome includes the fifth pediger. The position of the oral papilla has been used as an accessory character to separate the genera. In general it is located anteriorly (less than 30% of way back along the ventral surface of the cephalothorax) in *Cymbasoma* and *Maemonstrilla*; it is closer to the middle of the cephalothorax in both *Monstrilla* and *Monstrillopsis*.

The antennules of female monstrilloids are formed of 4 segments, but in some species such as *M. elongata* Suárez-Morales, 1994 or *M. longiremis* Giesbrecht, 1893, segmentation is weak. The setation related to each segment is recognizable following the known patterns [34]; a detailed illustration of this pattern was published along with the upgraded descriptive standards for the group [34]. In some species, the antennules have additional constrictions and protuberances (*M. mariaeugeniae*) [41], deep ridges or scales (*M. spinosa* Park, 1967) [42], or a light reticulation (*Maemonstrilla spinicoxa* Grygier & Ohtsuka, 2008). There are four different kinds of armature elements found on monstrilloid antennules: spiniform setae, simple setae, aesthetascs, and branched setae. Branched setae are found exclusively on the distal segment of the antennules of males and females of many species; branching can be simple or complex. In some cases the largest setae can be associated with basal processes with spine patches as in *C. javense* (Isaac, 1974) [28] or pilose areas in *M. elongata* [22]. The overall reductions in the segmentation and number of setal elements have obscured the interpretation of homologies of these elements [22]. Males have 5-segmented antennules and show the same basic setation pattern known from the females at least on the first four segments [34]. The strongest modification of the male antennule is the distal geniculation, which includes a single segment bearing up to 12 setal elements, including branched and unmodified setae [38]. Some species of *Monstrilla*, such as *M. brasiliensis* Suárez-Morales & Dias, 2000 and *M. inserta* Suárez-Morales, 2001 have a peculiar distribution of the antennular armature, with the distal groups of elements of the distal segment clustered near the terminal end of the last segment and separated from the proximal elements by an elongation of the segment [43], [44].

There are four different morphological types of male antennules [22]: (1) lacking special modification of the distal segment, as in many species of *Monstrilla* or *Cymbasoma*, (2) with a hyaline process on the medial margin of this segment, which tapers distally to a curved tip, only known in *Monstrillopsis* [11], (3) with distal transverse rows of serrate ridges, exclusive to some species of *Monstrilla* [39], [45], and (4) with reduced, vestigial rows of ridges, as in previous pattern. The homologies of the setal elements of the distal antennular segment were described in a recent contribution [38].

Aside from the position of the oral papilla, the cephalothorax has a number of characters whose taxonomic relevance has yet to be explored. The paired scars on the anteroventral surface of the cephalothorax, also known as nipple-like processes, are variable in terms of position, size, and adjacent ornamentation (i.e. wrinkles or deep ridges). Also on the anteroventral surface of the cephalothorax, pilose patches or pustules (*M. brevicornis* Isaac, 1974, *M. pustulata* Suárez-Morales & Dias, 2001) or even large wart-like ornamentations (*M. inserta*) have been reported. The cephalothoracic cuticular ornamentation can be distinctive; a simple, light reticulation has been recorded in *Cymbasoma reticulatum* (Giesbrecht, 1892) and *Monstrilla reticulata* Davis, 1949 and a more complex, heavier pattern was described in some species of *Maemonstrilla* [6]. In *C. striatum* (Isaac, 1974) a wide fringe of cuticular striations runs around the body and covers almost half the length of the cephalothorax [28]. Some of these ornamentations are present also on the dorsal surface of the first urosomites. The pattern of cuticular pores and pit setae on the dorsal and lateral surfaces of the cephalothorax has been explored by SEM in species of *Maemonstrilla*, and less completely in other species by light microscopy [6]; the pattern appears to be, in general, homogeneous in the group, but current knowledge of inter- and intra-specific variability is still insufficient to propose its incorporation to the taxonomy of the group.

Monstrilloids have four pairs of swimming legs with a quite uniform structure. Both rami are always three-segmented, with a conservative ancestral spine and setal formula [22] and a reduction of the number of setae on the exopod of the first leg. Aside from general medial setal reduction in species of *Maemonstrilla*, only in *Monstrilla leucopis* Sars, 1921 has a reduction on the endopodal armature been observed, and just on the first leg of some individuals [46]. Also, this species has modified setae on the endopods of legs 2–4; stout, thick-walled setal elements clearly differing from regular setae and unique among the Monstrilloida. The surface of the swimming legs is usually naked, but in some species it is ornamented, mainly on the outer surface of both the coxa and the exopod. Patterns include the uniform coverage of denticles found in *Maemonstrilla hyottoko* or a combination of large spikes and patches of denticles described in *M. spinicoxa* [6]. A unique character present only in species of *Maemonstrilla* is the wide separation of the legs 1–4 from the longitudinal body axis; the intercoxal sclerites are low and wide, whereas they are usually subquadrate in most species. Presumedly, these features as well as the above-mentioned setal reduction are adaptations for subthoracic brooding [6].

The female fifth leg is one of the most relevant taxonomic characters in the Monstrilloida. The ancestral leg is biramous, each ramus being one-segmented. The maximum setation pattern of 3 exopodal and 2 endopodal setae is present only in some species of *Monstrilla*, such as *M. grandis*, *M. orcula*, and *M. cymbula* [22], [39]. A pattern of 3-1 is found also in *Monstrilla*, e.g. *M. brasiliensis*, *M. careli*, *M. humesi*, and *M. inserta* [43], [44], [47]. A 2-2 pattern has been described exclusively in a species of *Monstrilla* (*M. grygieri* Suárez-Morales, 2000). In *Cymbasoma*, the most frequent pattern includes a naked inner lobe and an outer lobe armed with 3 setae, as in the *C. longispinosum* species-group [32] and in *C. gigas* [29]; the inner lobe can be much reduced or absent, as in *C. boxshalli* and *C. striatus* [28], [44]. In *Monstrillopsis* the fifth leg exhibits different patterns, including (1) an unarmed inner lobe and an outer exopodal lobe bearing 3 setae, (2) inner lobe armed with a single seta, (3) inner lobe absent [11]. In *Maemonstrilla* the form of the fifth legs is homogeneous in most of the known species, a single, rod-like lobe with 2 distal setae, except for *M. turgida* Scott, with an inner naked lobe and an outer lobe armed with 3 setae [6]. Males of *Monstrilla*, *Cymbasoma* and *Monstrillopsis* lack fifth legs; in some *Monstrilla*, as in *M. conjunctiva* Giesbrecht, *M. grandis*, and *M. longiremis* the fifth legs are represented by single lobe armed with a distal seta.

Male monstrilloids have a copulatory organ on the genital somite; this is one of the most valuable taxonomic characters for distinguishing and recognizing male specimens of different species [25]. In general, the male organ is represented by a basal shaft with divergent distal processes (lappets). The shaft can be short and broad as in many species of *Cymbasoma* and *Monstrilla* or globose as is *M. wandeli* Park, 1967, *M. elongata*, and *M. spinosa* Park, 1967. An elongate, cylindrical shaft has been described only in species of *Monstrilla*, such as *Monstrilla reidae* Suárez-Morales, 1993, *M. bahiana* Suárez-Morales & Dias, 2001, and *M. globosa* Suárez-Morales, 2003. The lappets are usually subtriangular, leaf-like or thumb-like processes, but other shapes can be found: digitiform in *Monstrillopsis sarsi* Isaac, 1974, or spiniform in *Monstrilla longiremis* Giesbrecht, 1893. Lappets can have widely divergent rami as in *Monstrillopsis dubia* and *M. dubioides*. In *M. wandelii*, they are represented by a pair of chela-like processes inserted on the posterior surface of the shaft [42]. A similar pattern is known for *C. javense* and *M. arctica* Davis & Green, 1974, but the lappets are dagger-like and thumb-like, respectively and the shaft has terminal spiniform processes in both. In many species, such as *C. longispinosum* (Bourne, 1898), *C. bullatum* (A. Scott, 1909), *C. chelemense* Suárez-Morales & Escamilla, 1997, and *Monstrillopsis chathamensis* Suárez-Morales & Morales-Ramírez, 2009, lappets arise directly from a common reduced base on the genital somite. In some cases lappets have distinguishing ornamentations or processes. In *C. mcalicei* Suárez-Morales, 1996, the inner margin of the lappets is creased whereas both margins (inner, outer) are wrinkled in *C. rugosa* Davis, 1947. In *C. quadridens* the inner margin has a row of 4–6 denticles. In some other species lappets have spiniform processes near the insertion of the organ shaft (*Cymbasoma tenue* Isaac, 1974, *C. rochai* Suárez-Morales & Dias, 2001). The genital organ is connected to internal sperm ducts that open at its distal end [22].

The caudal rami bear a number of setae that is variable among the genera. Some species of *Monstrilla* have 5 or 6 setae, all female *Monstrillopsis* have 4 (except for *M. zernowi* Dolgopolskaya, 1948) [11], and all species of *Cymbasoma* show a further reduction with only three 3 caudal setae. In *Maemonstrilla*, all species uniformly have 6 caudal setae. Adult copepods usually bear 7 setae, but the maximum number found in adult monstrilloids is 6. By interpreting the ontogenetic trajectories of the caudal setae in the naupliar stage and in the adult, it has been suggested that the dorsal seta VII is always absent in this group [38] and that the early copepodite setation pattern remains unaltered to the adult stage. Of course, the identity of the missing seta can only be confirmed by comparison with adults of other copepod orders.

### Life Cycle

The life cycle of monstrilloid copepods corresponds to the protelean model, in which free-living adult forms emerge from endoparasitic juveniles. There appears to be only a single free swimming lecithotrophic naupliar stage; it probably has the task of selecting, locating, and attaching to the potential polychaete or molluscan host. The first mention of the naupliar stage of the Monstrilloida [48] included comments of reared eggs carried by an adult female of *Cymbasoma longispinosum*. Later on [16], a description and illustrations of the nauplius and the subsequent endoparasitic development of a species of *Haemocera* were provided. Almost a century after these findings, detailed SEM observations were made of the first nauplius stage of the Japanese species *Monstrilla hamatapex* [34]. Based on these new data the authors concluded that naupliar appendages have more segments and setal elements than previously reported. They observed similarities with the naupliar pattern of cyclopoids, poecilostomatoids, and also with some harpacticoids, but they recognized a divergence in the placement of the labrum and antennules, both set far from the anterior rim. The antennule retains many plesiomorphic characters, but the antennal precoxa, present in many copepod nauplii, is absent in monstrilloids. The two-segmented antennal endopod plus a reduced basis also diverges from most of the known patterns among the main orders of the Copepoda. Two of the main naupliar apomorphies of the monstrilloids are: a denticle and sensillum at midlength of the second endopodal segment of the antenna vs. the plesiomorphic condition of 1 or 2 setae on the same place, and the presence of a claw-like seta on this antennal endopod vs. 1 or 2 setae representing the plesiomorphic condition. A later comparison of the nauplius of *Monstrilla hamatapex* to that of *Maemonstrilla okame* [6] showed them to be quite similar except for details of the antennules and mandibles.

The infection process has been described only partially in the Monstrilloida [1], [8] but it is presumed that it does not differ much from the strategies shown by other parasitic copepods. The mandibles of this first naupliar stage have a pair of terminal claws which, together with the antennae, would be efficient tools to attach themselves to the potential host and then burrow into its body. The first nauplius is the only naupliar instar that is comparable to other copepod naupliae because after infection, the endoparasitic naupliar stages undergo a deep transformation to a cigar-shaped “pupa” that bears little resemblance to other crustacean larvae [13], [22], [34]. After infection, the copepod develops within the host (usually a benthic polychaete) and forms a protective sheath around the body. Two antero-ventral root-like processes allow the parasite to absorb nourishment from the host. At the last juvenile phase, the copepod leaves the host through the body wall of the host as a last preadult with fully formed but structurally rather simple appendages and appendage armature. After a final moult it becomes a reproductive adult.

Other invertebrate taxa (e.g. platyhelminths, nematodes, crustaceans) have members with protelean life cycles, but among the Copepoda, only monstrilloids and thaumatopsyllioids have this kind of life cycle. A recent phylogenetic analysis [38] suggests that there was a common ectoparasitic ancestor of monstrilloids and caligiform taxa and that these lineages diverged by shifting hosts; in this process monstrilloids became parasites of invertebrates (vs. vertebrate hosts of caligids), acquired a transformed nauplii, lost their mouthparts; while the larval stages became endoparasitic, and adults became a free-living reproductive and dispersal stage.

### Biogeography

Based on worldwide data from different sources [8], [18], [19], [24], [Suárez-Morales unpubl. data], a general summary of the records of species of monstrilloids has been assembled. As a result, an indication of the regions in which the group is best and least known became clear. Records for which locales are unknown, or those repeated by the same author in reference to the same site, or those with a marginal reference to the group, were not considered. All in all, approximately 500 historical records of monstrilloid species were reviewed. Among them, about 45% pertained to the Northeast Atlantic, comprising European waters; almost 17% were from the Northwestern Atlantic, including temperate and tropical latitudes, 11% were from the Mediterranean, 8% from the tropical Indo-Pacific and also 8% from Asian waters of the Pacific (including the China and Japan seas). The least surveyed regions, with less than 3% each include the Southeastern and Southwestern Atlantic, and the Australian waters. The Southern Ocean, including the Antarctic Convergence, is also among these large geographic areas for which the monstrilloid copepod fauna is still poorly known. Around South America there are only a few records of Monstrilloida from high latitudes [11], [49]–[54], and only two species have been hitherto reported elsewhere from Subantarctic waters [24], [55].

There have been few attempts to explore the biogeography of this group. This kind of analysis must be based on a complete, reliable set of distributional data and this condition is not met among the Monstrilloida. The taxonomic problems recounted above have led to many unlikely records and also improbable cosmopolitan distributions. For instance, *Monstrilla anglica*, first known from different parts of Europe was later recorded from Java, Vietnam, and Florida; *Monstrilla danae* has been reported from Helgoland and adjacent areas of cold temperate latitudes and also from Vietnam. Given such conditions, it seemed more efficient and informative to start by analyzing the smaller genera. The known species of *Monstrillopsis* are distributed mainly in temperate and cold latitudes of both hemispheres [11]: *M. dubia* (Scott, 1904), not anymore regarded as a cosmopolitan form, is restricted to Scotland (60°N). *Monstrillopsis zernowi* Dolgopolskaya, 1948 is known only from the Black Sea (43°N) where it was originally described, *M. sarsi* Isaac, 1974 is distributed in English waters (54°N), *M. fosshageni* Suárez-Morales and Dias, 2001 in Brazil (20°S), *M. dubioides* Suárez-Morales, 2004 in Norway (62°N), *M. ferrarii* Suárez-Morales and Ivanenko in the White Sea, Arctic Ocean (66°N), *M. chilensis* off Chile (33°S), and *M. igniterra* in the Southern Ocean (55°S). Only two species of *Monstrillopsis* have been recorded from subtropical areas (*M. fosshageni and M.* cf. *dubia*) [11], [53]. Even in this small genus there are problems to solve before completing the distributional patterns of some species. All European and North American records of *M. dubia* should be revised, as some may pertain to closely related species of the Northern Hemisphere, probably resembling *M. ferrarii* or *M. dubioides* [23]. Records of *M. dubia* from the Southern Hemisphere could refer to *M. chilensis*, *M. igniterra* [11], or undescribed taxa. The recently described genus *Maemonstrilla* has an Indo-West Pacific distribution. Most species have been found in Japanese coral reef areas in the Ryukyu Islands, but *M. turgida* has been known to occur in the Ceram Sea, Indonesia, and also in Indian waters and the South China Sea. Also, *M. longipes* is known from Indonesia, as well as supposedly the Nicobars, Singapore, and the Red Sea. With the current information on this genus, there is really very little to say about its biogeography other than it tends to occur in the Indo-West Pacific and that it is possibly connected with coral reef environments.

Based on data from different sources [6], [18], [24], [27], [55], [56], [Suárez-Morales unpubl. data], the distribution of the diversity of the Monstrilloida in different regions of the oceans [sensu 18] was analyzed. The regions with the highest species richness are the European waters of the North Atlantic (32 species), followed by the Caribbean Sea and Gulf of Mexico (24), the Mediterranean and the Black Sea (19), Indonesia-Malaysia-Philippines region (17), Japan Seas (17), and the Brazil-Argentine area (16). The genus *Monstrilla* appears to be highly speciose in the Caribbean Sea-Gulf of Mexico area (15 species), in European waters (14), and the Indonesian area (10). *Cymbasoma* appears to be more diverse in Europe than in the Caribbean-Gulf of Mexico (14 species vs. 7). *Monstrilla* and *Cymbasoma* are almost equally species-rich in European waters (14-14), the Mediterranean (9-8), Japanese waters (4-5), and in the Indian Ocean (7-7). The former genus is more speciose than the latter in the Caribbean-Gulf of Mexico (15-7) and in the Indonesia-Malaysie-Philippines region (10-5).

### Additional Remarks

The Monstrilloida have been known to infest several species of polychaetes, but also pyramidellid [17] and vermetid gastropod molluscs [38]. Recently, a species of *Monstrilla* was recorded infecting the mantle of the commercially valuable bivalve, the brown mussel *Perna perna* in Brazil [57]. It was found with a high prevalence in this cultured population and a recent episode of mortality of this mussel (almost 20%) was partially attributed to this *Monstrilla*. If correct, this would be the first report of a monstrilloid's negative effect on a population of commercially valuable invertebrates. The high prevalence (25.6%) of *Monstrilla* in *P. perna* culture fields in Brazil is most probably a result of the artificial concentration of potential hosts for larval infective monstrilloids. The availability of hosts is a key factor in determining the adult populations of monstrilloids along the water column, particularly in shallow water environments [29], so a culture field of potential hosts represents a highly favourable situation for monstrilloids. The presence of the copepod larvae severely damages the mantle's tissue of the brown mussel; it causes hyperplasia and a strong inflammatory response from the host, including haemocyte migration to the mantle area in which the copepodite is lodged.

Not much information is available concerning population-level effects on other hosts. In a natural population of the gastropod *O. scalaris*, a relatively low prevalence (2%, 4 out of 200 individuals examined) of these parasites was reported [17]. The author of that report reared these juvenile specimens to adulthood and identified them as *M. helgolandica*. Off the coast of California, a 1% prevalence of *M. capitellicola* Hartman, 1964 was reported from the polychaete *Capitella capitata oculata* [40].

Despite the fact that monstrilloids tend to be more diverse and abundant in coral reef situations [4], [58], they were not recognized as swarm formers. Recently, a natural aggregation of monstrilloids was observed in a Caribbean reef environment [29]. More than 800 individuals of a single species were collected during one ordinary plankton trawl. This aggregation of monstrilloids appears to be the one with the highest concentration ever recorded, even considering other studies employing light traps.

Monstrilloids have been reported to have seasonal peaks of abundance in the water column. In a Brazilian estuary they are most abundant during the dry season and they were absent during the rainy period [33]. A plankton survey off the coasts of Brazil [54] revealed that each of the main water masses (tropical, subtropical, Antarctic) present in the area can be roughly characterized by a defined assemblage of monstrilloid species. The knowledge on their distribution and abundance patterns of these copepods in the plankton needs to be studied further.

A huge amount of fundamental research is still pending in reference to this intriguing group. The strategy to follow is to keep upgrading the descriptive standards while continuing the revisionary work based on type specimens, but also to develop more experimental approaches. Such studies are needed to reveal additional information about the ontogeny of this group, in particular the details of the infection mechanism, the effects of the monstrilloids as parasites, the hatching process, and host specificity.
